# LIFETIME TRAUMA AND ALLOSTATIC LOAD: PSYCHOSOCIAL RESOURCES AS BUFFERS?

**DOI:** 10.1093/geroni/igae098.3213

**Published:** 2024-12-31

**Authors:** Mallory Bell, Wencheng Zhang, Kenneth Ferraro

**Affiliations:** Purdue University, West Lafayette, Indiana, United States; Purdue University, West Lafayette, Indiana, United States; Purdue University, West Lafayette, Indiana, United States

## Abstract

Although past studies report a relationship between stress and allostatic load, less research examines whether psychosocial resources buffer this relationship. Further, most studies examine allostatic load cross-sectionally. The current study uses sixes waves (2006-2016) of the Health and Retirement Study to examine the relationship between lifetime traumas and allostatic load over time. We also examine whether a variety of psychosocial resources, such as neighborhood cohesion, social support, and volunteering, help protect against the deleterious consequences of trauma on physiological dysregulation. Results indicate that higher lifetime trauma is associated with higher allostatic load. Allostatic load did not change over time Further, we found little evidence that psychosocial resources moderate the relationship between lifetime traumas and allostatic load.

